# Monetite vs. Brushite: Different Influences on Bone Cell Response Modulated by Strontium Functionalization

**DOI:** 10.3390/jfb13020065

**Published:** 2022-05-24

**Authors:** Elisa Boanini, Stefania Pagani, Matilde Tschon, Katia Rubini, Milena Fini, Adriana Bigi

**Affiliations:** 1 Department of Chemistry “Giacomo Ciamician”, University of Bologna, Via Selmi 2, 40126 Bologna, Italy; katia.rubini@unibo.it (K.R.); adriana.bigi@unibo.it (A.B.); 2 Complex Structure Surgical Sciences and Technologies, IRCCS Istituto Ortopedico Rizzoli, Via di Barbiano 1/10, 40136 Bologna, Italy; stefania.pagani@ior.it (S.P.); matilde.tschon@ior.it (M.T.); milena.fini@ior.it (M.F.)

**Keywords:** calcium phosphates, ion substitution, strontium, co-culture, osteoblast, human primary osteoclast

## Abstract

Monetite and brushite are regarded with increasing interest for the preparation of biomaterials for applications in the musculoskeletal system. Herein, we investigated the influence of strontium substitution in the structures of these two phosphates on bone cell response. To achieve this aim, co-cultures of human primary osteoclasts and human osteoblast-like MG63 cells were tested on strontium-substituted monetite and strontium-substituted brushite, as well as on monetite and brushite, as controls. In both structures, strontium substitution for calcium amounted to about 6 at% and provoked enlargement of the cell parameters and morphologic variations. Cumulative release in physiological solution increased linearly over time and was greater from brushite (up to about 160 and 560 mg/L at 14 days for Sr and Ca, respectively) than from monetite (up to about 90 and 250 mg/L at 14 days for Sr and Ca, respectively). The increasing viability of osteoblast-like cells over time, with the different expression level of some typical bone markers, indicates a more pronounced trigger toward osteoblast differentiation and osteoclast inhibition by brushite materials. In particular, the inhibition of cathepsin K and tartrate-resistant acid phosphatase at the gene and morphological levels suggests strontium-substituted brushite can be applied in diseases characterized by excessive bone resorption.

## 1. Introduction

Monetite (DCPA) and brushite (DCPD) are the anhydrous (CaHPO_4_) and di-hydrated (CaHPO_4_·2H_2_O) form of dicalcium phosphate, respectively. Although these compounds have not been reported in physiological calcification [1], they are widely employed in calcium phosphate bone cements, coatings of metallic implants and bone grafts [2,3,4,5,6]. One of the main advantages of DCPA and DCPD in comparison to the more renowned hydroxyapatite, octacalcium phosphate and tricalcium phosphate, is their much better resorption, which is related to their higher solubility [1,2]. Moreover, they are good osteoconductive and osteoinductive materials [3,7,8,9,10,11].

DCPD exhibits a monoclinic structure (*I*a space group), is stable at pH < 6.5 and loses its two structural water molecules to yield DCPA when heated at about 190 °C [12]. The structure of DCPA is triclinic, and its stable α form at room temperature crystallizes in the P-1 group [13].

The study of ionic substitution in calcium orthophosphates is of remarkable interest, both because of the great variety of foreign ions associated with the mineral phase of the hard tissues of vertebrates and because of the important biological role played by many of these ions [14,15,16]. In particular, a great deal of research has addressed the strontium ion, which has been shown to promote bone growth and osteogenesis [17,18]. It stimulates osteoblast proliferation and differentiation, but exerts an inhibitory role on osteoclastogenesis and osteoclast activity, also when associated to calcium phosphates [19,20,21,22]. On this basis, calcium phosphates have been proposed as delivery systems for the local release of strontium ion in tissues affected by pathologies involving abnormally high bone resorption. We have recently demonstrated that strontium can substitute calcium in the structure of monetite in the whole range of composition [23], with 0 to 100% strontium atoms replacing calcium: α-SrHPO_4_ can be obtained, which is isomorphous with α-CaHPO_4_ [24]. In contrast, the brushite structure can host just a limited amount of strontium, replacing calcium at up to about 38 at% [25]. Tamimi et al. [7] reported that monetite ceramic induces a higher level of osteogenic gene expression of bone marrow cells in comparison to brushite ceramic; however, the same authors underline that these results could be due to the different solubility, surface area and porosity of the two ceramics. Moreover, strontium-containing monetite coating on metallic substrates was shown to improve the biological response. The substitution of strontium for calcium in monetite was confirmed by significant variations in lattice parameters by XRD analysis and Rietveld refinement [26].

In this study, we analyzed the effect of strontium substitution in the structures of monetite and brushite on the response of bone cells. To this aim, we compared the results of co-cultures of human primary osteoclast and human osteoblast-like cells MG63 on Sr-substituted DCPA (SrDCPA) and Sr-substituted-DCPD (SrDCPD), using DCPA and DCPD as controls. The amount of strontium substitution for calcium was chosen on the basis of previous positive results obtained for different phosphates in vivo [21,22].

## 2. Materials and Methods

### 2.1. Synthesis and Physicochemical Characterization

Synthesis of DCPA crystals was carried out by dropwise addition (2 mL/min) of 50 mL of 0.65 M (NH_4_)_2_HPO_4_ solution to 50 mL of 1.08 M Ca(NO_3_)_2_·4H_2_O solution at 90 °C. The precipitate was maintained in contact with the reaction solution for 1 h at 90 °C under stirring, then centrifuged at 10,000 rpm for 10 min, washed twice with distilled water and dried at 37 °C. SrDCPA was synthesized following the same procedure, but replacing Ca(NO_3_)_2_·4H_2_O solution with a solution containing 0.972 M Ca(NO_3_)_2_·4H_2_O and 0.108 M Sr(NO_3_)_2_. Total cation concentration was kept at 1.08 M; this solution contained 10 Sr atom%, calculated as ([Sr^2+^/(Ca^2+^ + Sr^2+^)]⋅100).

For the synthesis of DCPD crystals, 150 mL of a solution containing 5 mmol of Na_2_HPO_4_·12H_2_O and 5 mmol of NaH_2_PO_4_·H_2_O was heated at 37 °C and the pH was adjusted to 5 adding glacial CH_3_COOH. Afterwards, 50 mL of a 0.2 M Ca(CH_3_COO)_2_·H_2_O solution was added dropwise under stirring. The precipitate was maintained in contact with the reaction solution for 10 min at 37 °C under stirring, and then filtered, washed with distilled water and dried at 37 °C. SrDCPD was synthesized following the same procedure, but replacing Ca(CH_3_COO)_2_·H_2_O solution with a solution containing 0.18 M Ca(CH_3_COO)_2_·H_2_O and 0.02 M Sr(CH_3_COO)_2_∙½H_2_O. The total cation concentration was kept at 0.2 M and this solution contained 10 Sr atom%, calculated as ([Sr^2+^/(Ca^2+^ + Sr^2+^)]⋅100).

The content of Ca and Sr in the solid products was analyzed by ion chromatography (Dionex ICS-90). Solid samples were previously dissolved in 0.1 M HCl.

X-ray diffraction analysis was carried out by means of a PANalytical X’Pert PRO powder diffractometer equipped with a fast X’Celerator detector (Cu Kα; 40 mA, 40 kV). The 2θ range was investigated from 10 to 60 degrees with a step size of 0.05 and time/step of 200 s. Data used for cell parameters calculations were processed with HighScore Plus software package (PANalytical).

In vitro tests were performed on disk-shaped samples (Ø = 6.0 mm). Each disk was prepared by pressing 40 mg of powder into cylindrical moulds by using a standard evacuable pellet die (Hellma), and sterilized using gamma rays (Cobalt-60) at a dose of 25 kGy.

Morphological investigation of the as-synthesized crystals and of the disk-shaped samples surface was performed using a Zeiss Leo-1530 high-resolution scanning electron microscope operating at 1 kV (InLens detector). No sample coating was performed.

For AFM imaging, a Veeco Nanoscope 3D instrument was used. The samples were analyzed in tapping mode using an E scanner (maximum scan size 15 μm) and phosphorus (n)-doped silicon probes (spring constant 20–80 N/m; resonance frequency 250–290 kHz; nominal tip radius < 10 nm). Roughness parameters, namely arithmetic mean roughness (Ra), root-square roughness (Rq) and the vertical distance between the highest and lowest points within the evaluation length (Rt), were recorded.

Release tests of calcium and strontium from disk-shaped samples was performed by immersing 1 disk-shaped sample in 1 mL of physiological solution (NaCl 0.9%). Cumulative release was measured by refreshing the solution at selected times for up to 14 days and analyzing the collected solutions by an atomic emission spectrometer (MP-AES Agilent 4210). This analysis was performed in triplicate.

### 2.2. In Vitro Co-Culture Model

Biological novelties introduced by these materials were tested by a bi-culture consisting of human primary osteoclasts (OC), obtained by differentiation of blood mononuclear cells, and human osteoblast-like MG63 cells (Istituto Zooprofilattico Sperimentale IZSBS, Brescia, Italy).

Mononuclear cells were isolated from peripheral human buffy coat of a healthy adult male donor (Ethics Committee, CE AVEC; approval n. 191/2019/Sper/IOR, 04/19), by density gradient centrifugation, as previously reported [27]. The obtained cells were counted, seeded in a 24-well plate at the density of 2 × 10^5^ cells/cm^2^ and cultured in osteoclast differentiating medium: Dulbecco’s modified Eagle medium (DMEM) supplemented with 10% FCS and antibiotics (100 U/mL penicillin, 100 µg/mL streptomycin), macrophage colony-stimulating factor (MCSF, 25 ng/mL) and receptor activator for NFkB ligand (RANKL, 30 ng/mL). Mononuclear cells were allowed to remain for 1 week in the appropriate medium to activate osteoclast differentiation, and frequently monitored under a microscope to determine adhesion and morphology [27].

MG63 cells were previously expanded in DMEM supplemented with 10% FCS, antibiotics (100 U/mL penicillin, 100 µg/mL streptomycin), β-glycerolphosphate (10^−4^ M) and ascorbic acid (50 µg/mL), then counted in a Neubauer chamber and then 5 × 10^4^ cells were seeded dropwise on the different samples (DCPA, SrDCPA, DCPD, SrDCPD). After seeding, cell suspension was left for 2 h on the substrates in a minimal volume, until the final volume of culture medium was added, to avoid cell dispersion and allow desired adhesion to materials.

This seeding of MG63 cells was carried out on the seventh day of OC differentiation, but in a separate well plate. When OBs were properly attached to the materials, the samples were moved to the same wells as the OCs, thus assembling the cultures (T_0_).

CTR condition was set up by seeding mononuclear cells on the well-plate bottom layer and MG63 on the culture insert (Millicel 0.4 μm pore size, PCF 12 mm diameter, Millipore, Tullagreen, Carrigtwohill, Co., Cork, Ireland), without materials.

### 2.3. Cell Viability

Cell viability was quantified at 7 and 14 days on the disassembled cultures, obtained by transferring samples seeded with OB in empty wells, in order to appreciate the contribution of each cell type. Alamar blue dye (Serotec, Oxford, UK) was added to OCs and OBs (1:10 *v*/*v*), both in presence of materials and in CTR condition, and incubated for 4 h at 37 °C. This non-toxic reagent allows evaluation of the cell activity on the same culture at different endpoints by the chemical reduction of its main component (resazurin) into resorufin in the mitochondria of living cells. The fluorescent product was quantified at 530ex–590em nm wavelengths using a microplate reader (VICTOR X2030, Perkin Elmer, Milano, Italy) and expressed as relative fluorescence units (RFU).

### 2.4. Osteoclast Differentiation

To assess the differentiation of the mononucleated cells to OC lineage, as well as the response of OC to the materials, TRAP (tartrate-resistant acid phosphatase) staining was performed after 7 and 14 d of co-culture, according to manufacturer’s instructions (SIGMA, Buchs, Switzerland). The positively stained cells showed different red intensity and multinucleation.

### 2.5. Gene Expression

The expression of some typical genes of OC differentiation and OB activity was evaluated after 14 d of coculture. Total RNA was extracted separately by OCs seeded on the well bottom and OBs seeded on the scaffolds, as well as by CTR condition, using the commercial RNeasy Mini Kit (Purelink™ RNA miniKit, Ambion by Life Technologies, Carlsbad, CA, USA). RNA was then quantified by a NANODROP spectrophotometer (NANODROP 2720 Thermal Cycler, Applied Biosystem, Life Technologies Italia, Monza, Italy) and reverse transcribed using the Superscript Vilo cDNA synthesis kit (Life Technologies, Carlsbad, CA, USA). Ten nanograms of cDNA was tested in duplicate for each sample.

Gene expression was evaluated by semiquantitative PCR analysis using the SYBR green PCR kit (QIAGEN GmbH, Hilden, Germany) in a Light Cycler 2.0 Instrument (Roche Diagnostics, GmbH, Manheim, Germany). The protocol included a denaturation step of 95 °C for 15 min, 25 to 40 cycles of amplification (95 °C for 15″, appropriate annealing temperature for each target, for 20″, and 72 °C for 20″) and a melting curve analysis to check for amplicon specificity. The following primer sets were used: *GAPDH* (forward: 5′-TGGTATCGTGGAAGGACTCA-3′, reverse: 5′-GCAGGGATGATGTTCTGGA-3′), *ALPL* (QuantiTect Primer Assay Hs_ALPL_1_SG), *COL1a1* (QuantiTect Primer Assay (Qiagen) Hs_COL1A1_1_SG), *CTSK* (forward: 5′-CAGACAACAGATTTCCATCAGC-3′, reverse: 5′-CTTCTTCCATAGCTCCCAGTG-3′), *ACP5* (forward: 5′-GAAGCGCAGATAGCCGTT-3′, reverse: 5′-GGTCACTGCCTACCTGTG-3′). The mean threshold cycle was determined for each sample and used for the calculation of relative expression using the Livak method (2^−ΔΔCt^), with GAPDH as reference gene and CTR at 7 d as the calibrator [28].

*ALPL* (alias: Alkaline Phosphatase—ALP); *COL1A1* (alias: Collagen type I—Col1); *CTSK* (alias Cathepsin K—CTSK), *ACP-5* (alias Tartrate-resistant acid phosphatase—TRAP).

### 2.6. Immunoenzymatic Assays

At 3, 7 and 14 d of co-culture, cell supernatants were collected and centrifuged to remove particulates, if present, and maintained at −70 °C until evaluation of their content. Enzyme-linked immunosorbent assay (ELISA) was performed to quantify the release of two of the main osteogenic markers.

Briefly, 100 μL of cell culture supernatant was added to 96-well plates coated with antibody specific to human alkaline phosphatase (ALP; Fine Test, Fine Biotech Co., Ltd., Wuhan, China. Catalogue No: EH2618), or collagen type I (COLL1; BIOMATIK, Biomatik Corporation, Cambridge, ON, Canada. Catalogue: EKU03297) and the assays were performed according to manufacturer’s instructions. Finally, the concentration of the markers was calculated by reading the absorbance at 450 nm on a spectrophotometer (Micro Plate reader, Bio-Rad Laboratories, Hercules, CA, USA) and referring to a standard curve of antigen concentration.

### 2.7. Statistical Analysis

Statistical evaluation of data was performed using the software package SPSS/PC+ Statistics 23.0 (SPSS Inc., Chicago, IL, USA). The results presented are the mean of six independent values. Data are reported as mean ± standard deviation (SD) at a significance level of *p* < 0.05. After having verified normal distribution and homogeneity of variance, one-way ANOVA was performed for comparison among groups. Finally, a post hoc multiple comparison test (Tahmane) was performed to detect significant differences among groups for gene expression.

## 3. Results and Discussion

### 3.1. Materials Synthesis and Characterization

**Figure 1:**
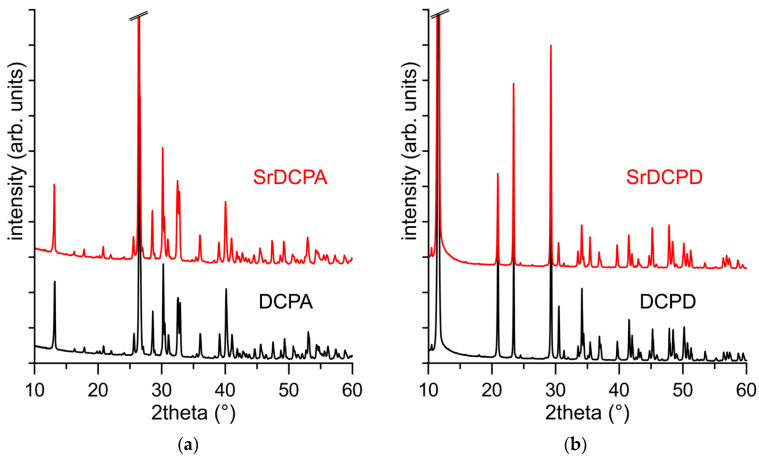
X-ray diffraction patterns of (**a**) DCPA, SrDCPA, and (**b**) DCPD, SrDCPD.

Sr-substituted DCPA and Sr-substituted-DCPD were prepared using the same strategy as the syntheses of DCPA and DCPD, respectively, but in the presence of strontium ions in solution corresponding to a stoichiometric amount of 10 at%. The products, SrDCPA and SrDCPD, exhibit X-ray diffraction patterns quite similar to those characteristic of pure monetite and brushite, as shown in Figure 1.

However, a close inspection of the patterns reveals that the presence of Sr provokes a modest shift of the diffraction peaks at lower angles in comparison to those of the pure calcium phosphates in both compounds. In agreement, the evaluation of the cell parameters of the different samples indicate a small but appreciable increase in the lattice constants due to the substitution of calcium with the bigger strontium ion, as reported in Table 1. The enlargement of the unit cells of DCPA and DCPD due to the incorporation of strontium is consistent with their content, determined through chemical analysis, which amounts to about 6 at% for both compounds (Table 1).

**Figure 2:**
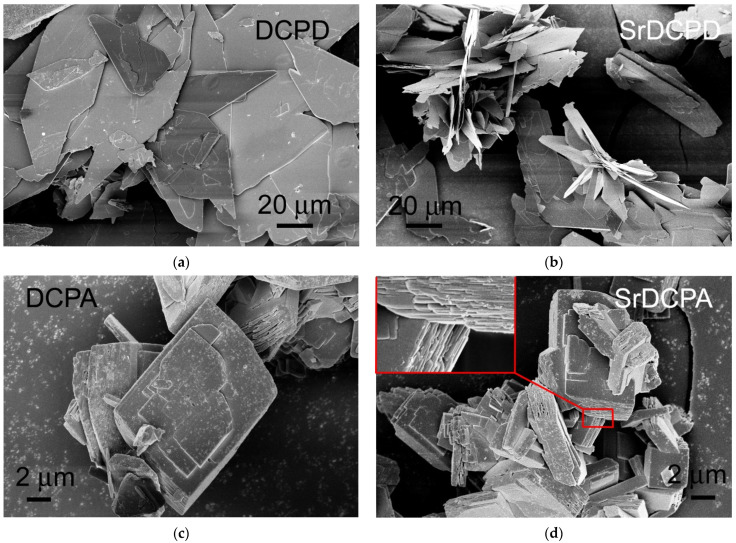
SEM images comparing morphology of (**a**) DCPD, (**b**) SrDCPD, (**c**) DCPA, and (**d**) SrDCPA crystals. The insert in the SrDCPA image displays its fine layered structure.

The scanning electron microscopy images of DCPA show that the compound is composed of crystals several microns wide, with a layered thickness (Figure 2). The corresponding product synthesized in the presence of Sr, shows a similar morphology, but the crystals are more indented and the layers become slightly thinner, as is clearly visible in the insert in SrDCPA image. The morphology of DCPD is quite different from DCPA; indeed, DCPD is composed of very big, plate-like crystals, characterized by large (0k0) faces. SrDCPD crystals preserve the overall plate-like shape of DCPD, but are slightly smaller in size and tend to aggregate, as shown in Figure 2.

**Figure 3:**
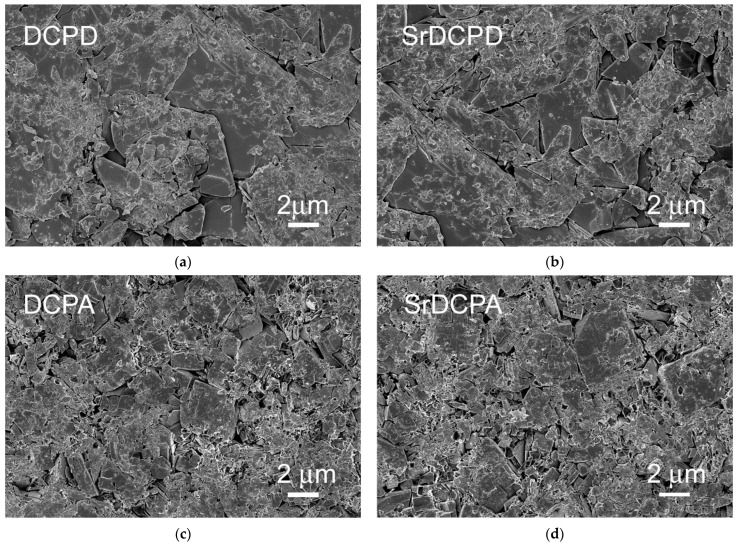
SEM images of the surfaces of disk-shaped samples of (**a**) DCPD, (**b**) SrDCPD, (**c**) DCPA and (**d**) SrDCPA used for in vitro tests. All images are the same magnification for direct comparison.

The variation in morphology induced by strontium in both compounds is no longer appreciable after the powders have been pressed into disks: the SEM images reported in Figure 3 show that the surfaces of the disks are similar, even if the presence of a few crystals retaining the pristine phosphate morphology is still appreciable.

**Figure 4:**
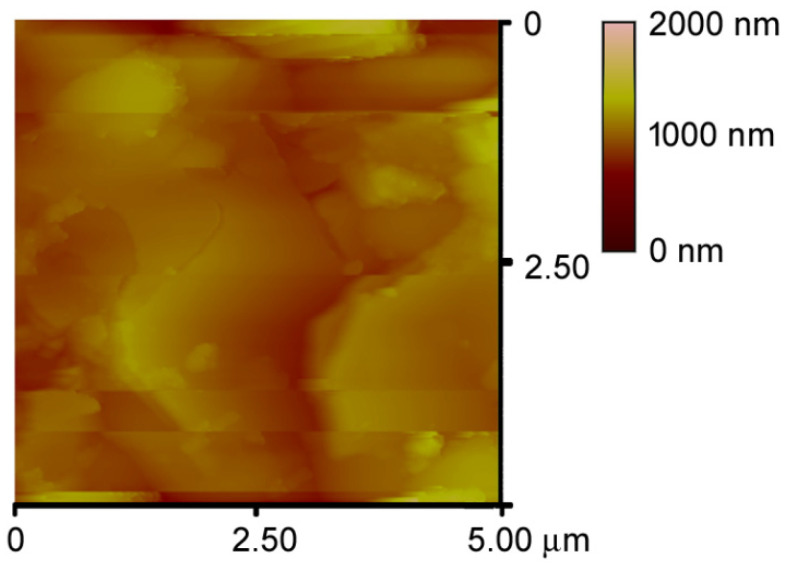
AFM image of the surface of disk-shaped sample of DCPD crystals.

All the surfaces are quite rough and difficult to analyze, as shown by the modest quality of the AFM image reported in Figure 4 for a DCPD disk. However, the roughness parameters of the different materials are similar, and assume the following mean values: Ra = 107.71 nm, Rq= 141.96 nm, Rt = 1.101 μm.

**Figure 5:**
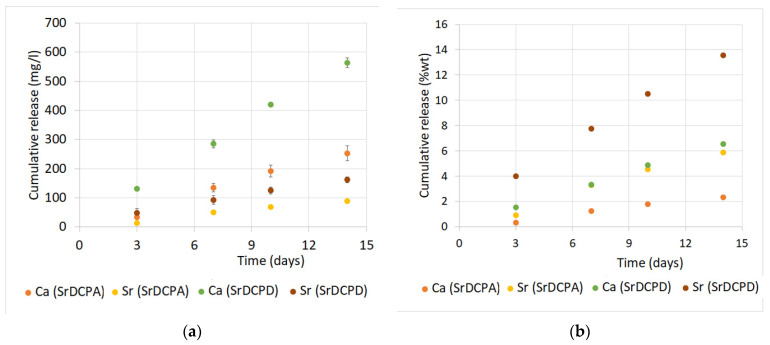
Calcium and strontium cumulative release from of SrDCPD and SrDCPA as a function of soaking time in physiological solution. Release is reported as (**a**) mg/L concentration detected in solution and (**b**) weight% calculated from initial amount.

Figure 5a reports Ca and Sr cumulative release from SrDCPA and SrDCPD in physiological solution. The release was investigated from disk-shaped samples, as for those utilized for cell co-cultures, in dynamic conditions up to 14 days. The data indicate that both Ca and Sr release increase linearly with time. The concentration of calcium measured in solution after 3 days was about 35 and 130 mg/L with SrDCPA and SrDCPD, respectively, and reached values of about 250 and 560 mg/L after 14 days. The first releases measured for Sr after 3 days in physiological solution amounted to about 15 and 50 mg/L from SrDCPA and SrDCPD, respectively, and increased over time up to about 90 and 160 mg/L. The greater ionic release from SrDCPD in comparison to SrDCPA is consistent with the greater solubility of brushite than monetite. A rough estimate of the cumulative release as percentage of the initial content (Figure 5b) indicates that the amount of Sr released in 14 days from SrDCPA and SrDCPD corresponds to about 6 wt% and 14 wt%, respectively. At the same time, Ca release amounts to about 2 wt% and 6.5 wt%. The sustained release should ensure a continuous local presence of the released ions for a long period of time.

### 3.2. Cellular Tests

#### 3.2.1. Cell Viability and Morphology

**Figure 6:**
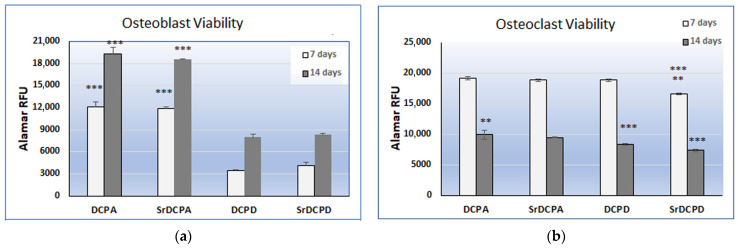
Cell viability determined by Alamar blue staining of osteoblasts (**a**) and osteoclasts (**b**) grown in co-culture on biomaterials after 7 days (light bars) and 14 days (dark bars). The results are given as relative fluorescent units and statistical analysis is reported in the graphs (** *p* < 0.005; *** *p* < 0.0005). (**a**) Osteoblast viability: 7d: *** DCPA vs. DCPD, SrDCPD; *** SrDCPA vs. DCPD, SrDCPD; 14d: *** DCPA vs. DCPD, SrDCPD; *** SrDCPA vs. DCPD, SrDCPD. (**b**) Osteoclast viability: 7d: *** SrDCPD vs. DCPA; ** SrDCPD vs. SrDCPA, DPCD; 14d: ** DCPA vs. SrDCPA; *** DCPD vs. DCPA, SrDCPA; *** SrDCPD vs. DCPA, SrDCPA, DCPD.

In order to evaluate the effects of SrDCPA and SrDCPD on the main bone cells, different biological tests have been performed. Initially, cell viability on the single co-culture components was evaluated; in general, a clear difference between DCPA and DCPD materials emerged in the OB graphs, where the values increased in the time. In contrast, OC viability decreased between 7 and 14 days, and only slight differences among the materials were deduced from the data. OBs grown on DCPA and SrDCPA had significantly viability than those grown on DCPD and SrDCPD, at both 7 and 14 days, but no differences were observed between DCPA and SrDCPA, or between DCPD and SrDCPD. For OB viability, the main difference seemed to be dependent on the phosphate type, regardless of the Sr addition (Figure 6a).

In general, the behavior of OCs was more similar on the different materials, but, in particular, they showed the lowest viability at 7 and 14 days when in contact with SrDCPD. After 14 days, SrDCPA had lower viability than DCPA, thus revealing a role of the Sr addition on OC. Moreover, DCPD exhibited lower viability than DCPA and SrDCPA, confirming the better influence of DCPA than DCPD scaffolds on cell viability (Figure 6b).

#### 3.2.2. Gene Expression

**Figure 7:**
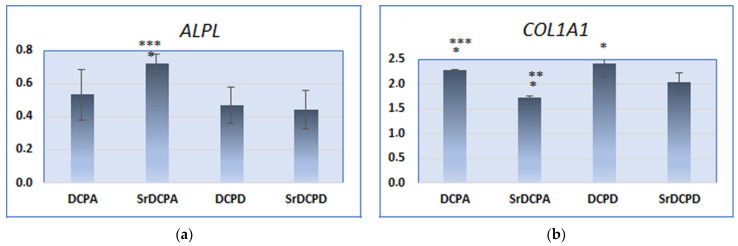

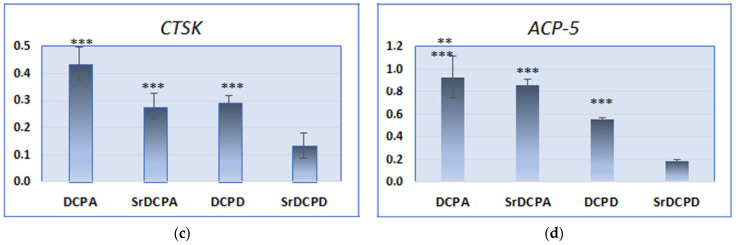
Gene expression of *ALPL* (**a**), *COL1A1* (**b**), *CTSK* (**c**) and *ACP-5* (**d**) after 14 days of co-culture of OB and OC in presence of the samples. Statistical analysis is reported in the figure (* *p* < 0.05, ** *p* < 0.005, *** *p* < 0.0005). ***ALPL***: * SrDCPA vs. DCPA; *** SrDCPA vs. SrDCPD; ***COL1A1***: *** DCPA vs. SrDCPA; * DCPA vs. SrDCPD; ** SrDCPA vs. DCPD; * SrDCPA vs. SrDCPD; * DCPD vs. SrDCPD; ***CTSK:*** *** DCPA vs. SrDCPA, DPCD, SrDCPD; *** SrDCPA vs. SrDCPD; *** DCPD vs. SrDCPD; ***ACP-5***: ** DCPA vs. DPCD, *** DCPA vs. SrDCPD; *** SrDCPA vs. DCPD, SrDCPD; *** DCPD vs. SrDCPD.

After 14 days of culture, the expression of *ALPL* and *COL1A1* was stimulated differently in the different samples: interestingly, OB seeded on SrDCPA seemed to be in an earlier state of activity, which might be related to its lower ionic release. In fact, SrDCPA resulted in higher *ALPL* expression than DCPA and SrDCPD, although the opposite situation was observed for *COL1A1*, with significantly lower expression stimulated by SrDCPA than by the other materials (Figure 7a,b). OBs seeded on all materials expressed less *ALPL* but more *COL1A1* than CTR (considered as 1 or reference group, not shown), thus helping to explain the state of activity of OBs after 14 d of culture, and, in particular, the positive effect of materials on bone synthesis. Furthermore, both Sr-doped materials had decreased *COL1A1* expression compared to the corresponding materials without Sr (Figure 7b).

**Figure 8:**
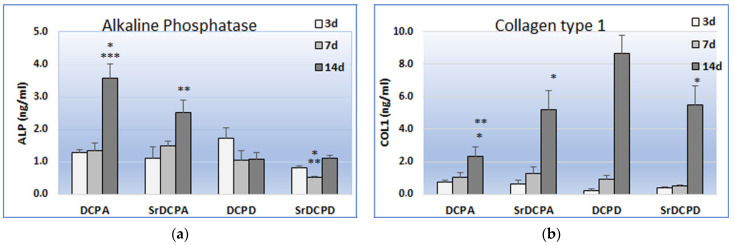
Release of ALP (**a**) and COL1 (**b**), after 3, 7 and 14 d of coculture of OBs and OCs in presence of the samples. Statistical analysis is reported in the figure (* *p* < 0.05, ** *p* < 0.005, *** *p* < 0.0005). **ALP***:* no significant differences at 3 days; 7d: * SrDCPD vs. DCPA, ** SrDCPD vs. SrDCPA; 14d: * DCPA vs. SrDCPA, *** DCPA vs. DCPD, SrDCPD; ** SrDCPA vs. DCPD, SrDCPD; **COL1**: 14d: ** DCPA vs. DCPD, * DCPA vs. SrDCPD, * SrDCPA vs. DCPD, * DCPD vs. SrDCPD.

The results obtained for protein quantification in the supernatant (Figure 8) indicated significant differences among the materials, particularly after 14 d of culture, thus making an interesting comparison with the observations of gene expression at the same timepoint. Furthermore, the strong increase in COL1 release between 7 and 14 d by OB on all materials revealed the second endpoint as important moment in matrix synthesis. Notoriously, the main role of OBs is to secrete bone ECM proteins, such as type I collagen, in a coordinated framework of gene activation. During the differentiation to osteocytes, for example, the decrease in ALP production corresponds to an increase in osteocalcin [29].

DCPD appeared the most promising sample from this point of view, showing the most abundant release of COL1 in comparison to the other materials, including SrDCPD, as also observed by *COL1A1* expression. The discrepancy that is instead observed between gene expression and protein release regarding DCPA materials, which is the more abundant production of COL1 by OBs grown on SrDCPA than DCPA and in particular the more abundant release of COL1 on brushite (with and without Sr) than monetite could be interpreted as the different timing of osteoblast maturation. The study of Idowu et al. reported a comparison between hydroxyapatite and monetite in term of MSC behavior, clearly displaying the cell differentiation phases and the earlier staging of MSCs on monetite than hydroxyapatite [30].

**Figure 9:**
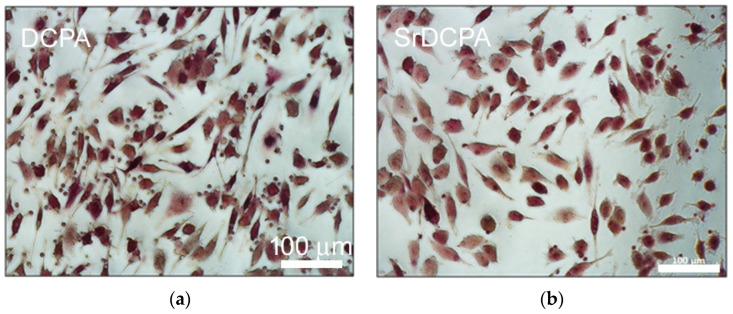

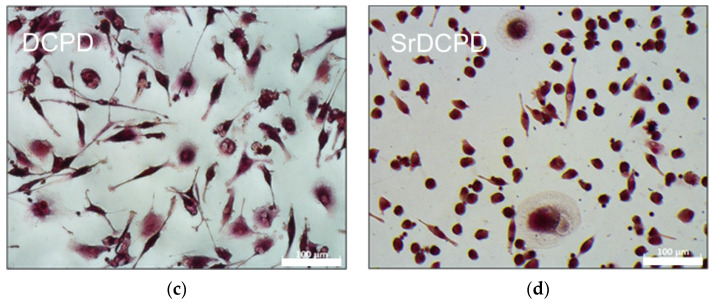
TRAP staining of osteoclasts after 14 days of co-culture with osteoblasts, in presence of the different materials (magn. 20×): (**a**) DCPA, (**b**) SrDCPA, (**c**) DCPD and (**d**) SrDCPD.

*CTSK* expression evaluated on the osteoclast cultures clearly showed that SrDCPD was the most influential in controlling this gene: the values of this group were also significantly lower than for SrDCPA (Figure 7c). It has already been known for several years that Sr negatively affects osteoclast activity, as indicated by the comparison between materials with and without this ion [31,32]. Similar results were obtained about *ACP5* expression, with a particularly pronounced difference between DCPD and SrDCPD (Figure 7d). For both these genes, DCPA revealed instead the highest values compared with all the other groups, thus showing its minor influence on OC activity. TRAP staining shown in Figure 9 described the morphology of OC in the presence of each material: in general, the cultures appeared poorly differentiated, particularly on SrDCPD, albeit with a few exceptions.

Collectively, these data can contribute to understanding how the synthesized materials interact with the main bone cells, stimulating or inhibiting their activities. The increase in osteoblast viability over time allows all materials to be considered suitable for the adhesion and growth of these cells. The higher values of SrDCPA and DCPA in term of viability could be sign of a greater proliferative stimulus in comparison to SrDCPD and DCPD, combined in fact with a condition of less osteoblastic differentiation, as shown by molecular biology and ELISA.

If, in healthy bone, the processes of synthesis and resorption are tightly kept in equilibrium as correct tissue remodeling, some particular disorders can negatively influence this delicate homeostasis, stimulating resorption and thus necessitating a decrease in osteoclast activity [29]. A stronger inhibitory action triggered by SrDCPD on OCs in comparison to SrDCPA, together with the observations about collagen stimulation indicate SrDCPD as more effective scaffold type for bone regeneration, especially in osteoporotic conditions. Nevertheless, it is appropriate to rate SrDCPA as an effective scaffold, but also to consider that probably it needs longer to achieve similar results.

A deeper analysis of gene expression performed by PCR, as well as of protein synthesis by ELISA or western blot at different timepoints could help to better understand the reaction of osteoblasts and osteoclasts to these materials and the timing of this biological process. Moreover, further investigations aimed at studying the interaction between these cell types in the presence of the materials would provide useful information to detail the influence of these calcium phosphates on osteoblast/osteoclast crosstalk.

## 4. Conclusions

The results of this study indicate that the behavior of bone cells depends on the type of calcium phosphate and is modulated by the presence of strontium inside the crystalline structure. Although the different phosphates examined exhibit different morphologies, the surfaces of the disk-shaped samples used for the cellular tests are quite similar, so the difference in data can be interpreted only on the basis of the different composition and solubility, which influence the observed sustained cumulative releases.

The evaluation of these materials in relation to the main bone cells and from the perspective of tissue homeostasis, revealed different effects of the studied calcium phosphates: monetite samples exert a stronger stimulus toward osteoblast proliferation and ALP production, whereas brushite materials are more effective in stimulating collagen and inhibiting osteoclast activity. The presence of strontium in the structures of these phosphates enhances these trends, confirming the beneficial role of this ion in promoting bone growth and inhibiting excessive bone resorption.

## Figures and Tables

**Table 1:** Crystalline lattice parameters and analytical content of strontium.

Sample	a (Å)	b (Å)	c (Å)	α (°)	β (°)	γ (°)	Sr Content (wt%)
DCPA	6.891(8)	6.639(5)	6.996(4)	96.15(5)	103.96(1)	88.51(1)	---
SrDCPA	6.927(9)	6.649(8)	7.014(5)	96.08(8)	104.07(1)	88.56(2)	5.8
DCPD	6.363(5)	15.189(4)	5.816(5)	---	118.58(1)	---	---
SrDCPD	6.385(5)	15.208(4)	5.825(4)	---	118.56(1)	---	5.6

## Data Availability

The data presented in this study are available on request from the corresponding author.
